# Higher levels of IgA and IgG at sepsis onset are associated with higher mortality: results from the Albumin Italian Outcome Sepsis (ALBIOS) trial

**DOI:** 10.1186/s13613-021-00952-z

**Published:** 2021-11-26

**Authors:** Laura Alagna, Jennifer M. T. A. Meessen, Giacomo Bellani, Daniela Albiero, Pietro Caironi, Irene Principale, Luigi Vivona, Giacomo Grasselli, Francesca Motta, Nicolò M. Agnelli, Vieri Parrini, Stefano Romagnoli, Roberto Keim, Francesca Di Marzo Capozzi, Fabio S. Taccone, Walter Taccone, Barbara Bottazzi, Alessandra Bandera, Andrea Cortegiani, Roberto Latini

**Affiliations:** 1 Department of Infectious Diseases, Fondazione IRCCS Ca’ Granda Ospedale Maggiore Policlinico, Milan, Italy; 2 Department of Cardiovascular Medicine, Institute for Pharmacological Research Mario Negri IRCCS, Via Mario Negri 2, 20156 Milan, Italy; 3 Department of Emergency and Intensive Care, San Gerardo Hospital, Via Giambattista Pergolesi 33, 20900 Monza, MB Italy; 4 Department of Medicine and Surgery, University of Milan-Bicocca, Via Cadore 48, 20900 Monza, MB Italy; 5 Department of Anesthesia and Critical Care, AOU S. Luigi Gonzaga, Orbassano, Italy; 6 Department of Oncology, University of Turin, Turin, Italy; 7 Department of Pathophysiology and Transplantation, Università Degli Studi di Milano, Milan, Italy; 8 Department of Anesthesia, Intensive Care and Emergency, Fondazione IRCCS Ca’ Granda Ospedale Maggiore Policlinico, Milan, Italy; 9 SOS Anesthesia and Reanimation, Ospedale del Mugello, Usl Toscana Centro, Borgo San Lorenzo, Florence, Italy; 10 Department of Health Science, Section of Anesthesia and Critical Care, University of Florence, Florence, Italy; 11 Department of Anesthesia and Critical Care, Azienda Ospedaliero-Universitaria Careggi, Florence, Italy; 12 UOC Anesthesia, Reanimation and Intensive Care, Ospedale Bolognini, Seriate, Bergamo Italy; 13 Futura Diagnostica srl, Avellino, Italy; 14 Department of Intensive Care, Université Libre de Bruxelles (ULB), Bruxelles, Belgium; 15 Department of Inflammation and Immunology, Humanitas Clinical and Research Centre – IRCCS, Milan, Italy; 16 Department of Surgical, Oncological and Oral Science (Di.Chir.On.S.), University of Palermo, Palermo, Italy; 17 Department of Anesthesia, Intensive Care and Emergency, Policlinico Paolo Giaccone, Palermo, Italy

**Keywords:** IgA, IgG, IgM Immunoglobulins, Mortality, Septic shock, Sepsis

## Abstract

**Background:**

The role of intravenous immunoglobulins (IVIG) during sepsis is controversial, as different trials on IVIG have observed inconsistent survival benefits. We aimed to elucidate the possible association and clinical significance between circulating levels of immunoglobulins.

**Methods:**

In a subset of 956 patients with severe sepsis and septic shock of the multicentre, open-label RCT ALBIOS, venous blood samples were serially collected 1, 2, and 7 days after enrolment (or at ICU discharge, whichever came first). IgA, IgG and IgM concentrations were assayed in all patients on day 1 and in a subgroup of 150 patients on days 2 and 7. Ig concentrations were measured employing a turbidimetric assay, OSR61171 system.

**Results:**

IgA on day 1 had a significant predictive value for both 28-day and 90-day mortality (28-day mortality, HR: 1.50 (95% CI 1.18–1.92); 90-day mortality, HR: 1.54 (95% CI 1.25–1.91)). IgG, but not IgM, on day 1 showed similar results for 28-day (HR 1.83 (95% CI 1.33–2.51) and 90-day mortality HR: 1.66 (95% CI 1.23–2.25)). In addition, lower levels of IgG but not of IgA and IgM, at day 1 were associated with significantly higher risk of secondary infections (533 [406–772] vs 600 [452–842] mg/dL, median [Q1–Q3], *p* = 0.007).

**Conclusions:**

In the largest cohort study of patients with severe sepsis or septic shock, we found that high levels of IgA and IgG on the first day of diagnosis were associated with a decreased 90-day survival. No association was found between IgM levels and survival. As such, the assessment of endogenous immunoglobulins could be a useful tool to identify septic patients at high risk of mortality.

*Trial registration* #NCT00707122, Clinicaltrial.gov, registered 30 June 2008

**Supplementary Information:**

The online version contains supplementary material available at 10.1186/s13613-021-00952-z.

## Background

Sepsis is one of the main causes of admission to intensive care unit (ICU) [1]. Both sepsis and septic shock are associated with an increased risk of death, with septic shock showing a mortality rate of more than 50% [2]. Cornerstones of the early-stage management of sepsis and septic shock are early recognition and resuscitation, source of infection control, adequate antibiotic therapy, achieving hemodynamic stability, and support of failing organs [3]. However, these measures and clinical therapies do not counter the inflammatory imbalance and the immunological dysregulation, including the subsequent prolonged immunosuppression, very often characterizing sepsis and septic shock [4, 5].

In patients suffering from these conditions, plasma concentration of all immunoglobulin classes has been reported as reduced [6, 7]. Moreover, low immunoglobulin levels during sepsis have been associated with poor outcome, suggesting the hypothesis that endogenous immunoglobulin replacement during sepsis may improve survival [8, 9].

The potential benefits of the use of intravenous immunoglobulins (IVIG) in severe infections is based on the two main functions of such therapy: antimicrobial activity and immunomodulatory effect [10]. Despite this rationale, the role of IVIG during sepsis is still controversial, as different randomized controlled trials (RCTs) have observed inconsistent survival benefits [11]. Moreover, even though low IgG has been reported, on average, in 58% of heterogeneous cohorts of patients on the day of diagnosis of sepsis, did not appear to be related to an increased risk of death [7]. Indeed, the nadir of immunoglobulin plasma concentration is often seen on day 3 following sepsis diagnosis [7, 12]. Moreover, other immunoglobulin subclasses (IgG1, IgM and IgA) have important immunomodulatory function and might also influence patients’ outcome. Finally, the association between low immunoglobulin concentrations and mortality is often observed in septic patients with less severe organ dysfunction [13], while the prognostic role of immunoglobulins remains unclear in most severe cases. These issues, therefore, suggest that our understanding of the mechanisms underlying the high prevalence of low immunoglobulins in sepsis and their impact on severity of the disease is incomplete.

We, therefore, aimed, in the present study, to elucidate the possible association and clinical significance between circulating levels of immunoglobulins, clinical characteristics and outcomes in a large cohort of critically ill patients with severe sepsis and septic shock.

## Methods

This was a secondary analysis of the Albumin Italian Outcome Sepsis (ALBIOS) trial. ALBIOS study was a multicentre, open-label, randomized, controlled trial on 1818 patients with severe sepsis or septic shock admitted to 100 Italian intensive care units (ICUs) to investigate the potential efficacy of albumin replacement (NCT00707122 clinicaltrial.gov). The study complied with the 1975 Declaration of Helsinki as revised in 2008 and was approved by the institutional review boards of each centre. Written informed consent or deferred consent was obtained from each participant, according to Italian legislation. Patients were randomly assigned to receive either 20% albumin and crystalloids or crystalloids alone for fluid resuscitation, from randomization until day 28 or discharge from the ICU, whichever came first. Primary outcome was 90 day mortality. Albumin administration was targeted to achieve a serum concentration of 30 g/L or more. Study design, inclusion and exclusion criteria, and main results have been published elsewhere [14].

### Definitions

In the original ALBIOS trial and for the purpose of this analysis, severe sepsis was defined as a proved or suspected infection in at least one site, two or more signs of systemic inflammatory reaction, and at least one severe, acute sepsis-related organ dysfunction [15]. Organ function was assessed on a daily basis with the use of the Sequential Organ Failure Assessment (SOFA) score [16], which rates for each of five organ sub-components (respiratory, coagulation, liver, cardiovascular and renal systems) from 0 to 4, with higher scores indicating a greater degree of organ dysfunction. Septic shock at the time of randomization was defined as severe sepsis with a SOFA score of ≥ 3 of the cardiovascular subcomponent.

### Assays

In a subset of 956 patients recruited in 40 centres that participated in a predefined biologic sub-study, venous blood samples were serially collected 1, 2, and 7 days after enrolment (or at ICU discharge, whichever came first). Blood samples were collected into ethylenediaminetetraacetic acid (EDTA) tubes, centrifuged, plasma stored at − 70 °C, and shipped on dry ice to a central repository at − 70 °C until assayed. All assays were performed by expert personnel, blinded to identity of plasma samples. Total immunoglobulin concentrations (IgG, IgA, and IgM) were assayed in all patients on day 1 and in a subgroup of 150 patients also on day 2 and 7. Ig concentrations were measured at Futura Diagnostica srl (Avellino, Italy) employing a turbidimetric assay, OSR61171 system (Beckman Coulter Inc., 250 S. Kraemer Blvd. Brea, CA 92821, USA). Calibration for the immunoglobulin procedure was performed every 90 days, using specific calibrators. Two levels of an appropriate immunology control material were tested daily as quality control, in accordance with regulatory requirements and laboratory standard procedure. The Limit of Quantitation (LOQ) for the immunoglobulin assay was 10 mg/dL.

In addition, data on inflammation biomarkers such as Presepsin [17], Pentraxin3 (PTX3) [18], Pro-protein convertase subtilisin/kexin 9 (PCSK9) [19], Resistin, myeloperoxidase (MPO) [20] and osteopontin (OPN) [21], already assayed in ALBIOS was included in this manuscript.

### Statistical analysis

Patients were divided into subgroups based on the levels of IgA, IgG and IgM on day 1 as follows [22–25]:

- “LOW” (i.e., IgA: < 70 mg/dL, IgG: < 700 mg/dL and IgM: < 40 mg/dL);
- “NORMAL” (i.e., IgA: 70–400 mg/dL, IgG: 700–1600 mg/dL and IgM: 40–230 mg/dL);
- “HIGH” (i.e., IgA: > 400 mg/dL, IgG > 1600 mg/dL and IgM > 230 mg/dL).

Patient characteristics were compared across these groups by means of Chi-square or Kruskal–Wallis test, as appropriate. Those variables found to be significantly different between these Ig-groups are then included in continuous multivariable linear regression models to assess which variables were independently associated with Ig-levels. Time course of immunoglobulins at day 1, 2 and 7 was assessed by means of repeated measures analysis, including a Greenhouse–Geisser correction if Mauchley’s test for sphericity was violated.

Kaplan–Meier curves were constructed to assess the association of the three Ig-groups and survival. Restricted cubic splines including three knots were created for 90-day mortality. Based upon these curves, only the values above the inflection point of the curve were included in multivariable survival analysis, relating z-transformed immunoglobulins to 28- and 90-day mortality by means of Cox proportional hazards models. These regression analyses were adjusted for the variables found to be independently associated with 28- and 90-mortality with a *p* < 0.0001. Association of Ig-levels and secondary infections was assessed by Kruskal–Wallis test. Data were analysed by means of SPSS Version 25 (IBM SPSS, Armonk, NY) and R-package “rms” (R foundation for statistical computing, Vienna, Austria).

## Results

### Study population

Concentrations on Day 1 of IgA, IgG and IgM were available for 956 patients. Demographic and clinical characteristics of the study population as divided according to immunoglobulin levels on day 1 are presented in Additional file 1: Tables S1, S2 and S3 for the total population. Characteristics of the 956 patients measured on day 1 after randomization are comparable with those of the total ALBIOS cohort are similar to those of the overall population [26]. More than half of the patients were in septic shock (56.3%), 90-day mortality was 38.6%. When stratifying patients according to immunoglobulin levels (i.e., low, normal and high), patients with the highest IgA, IgG or IgM had higher white blood cell count. Immunocompromised patients were more prevalent in the low range of IgA and in the high range of IgG. Prevalence of septic shock did not differ between ranges of IgA, G or M. Mortality was higher in the high range of IgA and IgG, but no difference was found for IgM. In 150 patients, immunoglobulin levels at day 2 and day 7 were also measured: the concentrations of all three immunoglobulins significantly increased over time (over time IgA *p* = 1.0 × 10^–18^; IgG *p* = 8.2 × 10^–10^; IgM *p* = 4.3 × 10^–10^ Additional file 1: Table S4). In the absence of formal statistical significance, concentrations of IgA and IgG, but not of IgM are slightly lower in albumin group at all three timepoints (Additional file 1: Table S4). A stronger correlation between IgA and IgG (Spearman *r*: 0.595) was observed as compared to IgG ad IgM (Spearman *r*: 0.484) and IgM and IgA (Spearman *r*: 0.320).

### IgA levels

On day 1, 85 out of 955 (8.9%) patients had LOW (median 49, IQR [34–59] mg/dL), 824 (86.3%) NORMAL (168.5 [120.3–221] mg/dL) and 46 (4.8%) HIGH circulating IgA levels (469, [443–527] mg/dL). Patients in the HIGH IgA subgroup were more frequently male and received more frequently albumin administration than others. None of the included inflammatory biomarkers was associated with IgA levels (Additional file 1: Table S1). Among comorbidities, the presence of previous immunosuppression and onco-hematologic disease was more often observed in the LOW IgA subgroup. In multivariable linear regression analyses, male sex, higher BMI, higher white blood cell count, cardiovascular comorbidities and study treatment allocation to crystalloids were all independently associated with higher IgA levels on day 1, whereas the presence of onco-hematologic pathologies was associated with lower IgA (Table 1).

**Table 1:** Multivariate linear regression analysis output for variables independently associated with IgA, IgG and IgM on day 1

Variable	B	95% CI	*p*
IgA			
Female sex	− 34.1	− 47.5: − 20.7	**7.0 × 10**^**–7**^
BMI	2.6	1.4: 3.8	**1.5 × 10**^**–5**^
White blood cell count at baseline	1.4	0.7: 2.1	**6.0 × 10**^**–5**^
CV comorbidities	28.6	11.2: 46.1	**0.001**
Randomized to crystalloids	19.4	6.3: 32.5	**0.004**
Onco-haematologic disease	− 48.0	− 87.0: − 9.1	**0.016**
Creatinine	− 3.5	− 7.6: 0.6	0.090
Immunocompromised state	− 18.3	− 40.0: 3.3	0.097
IgG			
Female sex	− 88.5	− 135.5: − 41.4	**2.4 × 10**^**–4**^
White blood cell count at baseline	4.2	1.7: 6.6	**0.001**
Randomized to crystalloids	73.7	27.5: 120.0	**0.002**
BMI	5.8	1.7: 10.0	**0.006**
Shock	− 55.1	− 101.8: − 8.4	**0.021**
Liver disease	188.5	− 3.5: 380.4	0.054
Heart rate	− 0.9	− 2.0: 0.3	0.127
Immunocompromised state	− 9.3	− 79.1: 60.6	0.794
IgM			
Age	− 0.6	− 0.9: − 0.2	**0.002**
Shock	− 14.4	− 25.3: − 3.6	**0.009**
Onco-haematologic disease	− 30.1	− 58.2: − 2.0	**0.036**
White blood cell count at baseline	0.4	− 0.1: 1.0	0.127
Mean arterial pressure	0.2	− 0.2: 0.5	0.329

Output for multivariable linear regression analysis with continuous Ig concentration as dependent variable

Included in the regression analyses are the variables which were significant in Additional file 1: Tables S1, S2 and S3. For IgG, AIDS was not included due to confounding by immuno-compromised state. Location of infection was not included in this analysis

### IgG levels

On day 1, 608 out of 956 (63.6%) patients had LOW (577 [372–572] mg/dL), 321 (33.6%) had NORMAL (900 [776–1076] mg/dL) and 27 (2.8%) HIGH IgG levels (1769 [1661–2003] mg/dL; Additional file 1: Table S2). Patients with HIGH IgG had more frequently a pulmonary infection than other groups. In the NORMAL subgroup, patients received less frequently albumin administration and had higher white blood cells count than other groups. In the LOW subgroup, patients were more frequently male, suffered more often from septic shock and presented more frequently immunodepression. PTX3 was significantly, inversely associated with IgG levels: it was highest at low levels of IgG. In the multivariable linear regression analysis, male sex, higher white blood cell count, study treatment allocation to crystalloids, higher BMI and shock were found to be independently associated with high IgG levels on day 1, whereas patients with shock as compared to patients with sepsis had lower IgG levels (Table 1).

### IgM levels

On day 1, 219 out of 956 (22.9%) patients had LOW (27 [1–33] mg/dL); 697 (72.9%) NORMAL (78 [56–115] mg/dL) and 40 (4.2%) HIGH IgM levels (337 [268–434] mg/dL; Additional file 1: Table S3). In the NORMAL subgroup, patients were younger. In the LOW subgroup, patients had more frequently septic shock and low mean arterial pressure, as well as lower white blood cell count as compared to the other groups. In addition, an inverse correlation was found for PTX3 and IgM. In the multivariable linear regression analysis, higher age, the presence of septic shock and onco-hematologic disease were found to be independently associated with decreased IgM levels on day 1 (Table 1).

### Immunoglobulin and survival

**Fig. 1:**
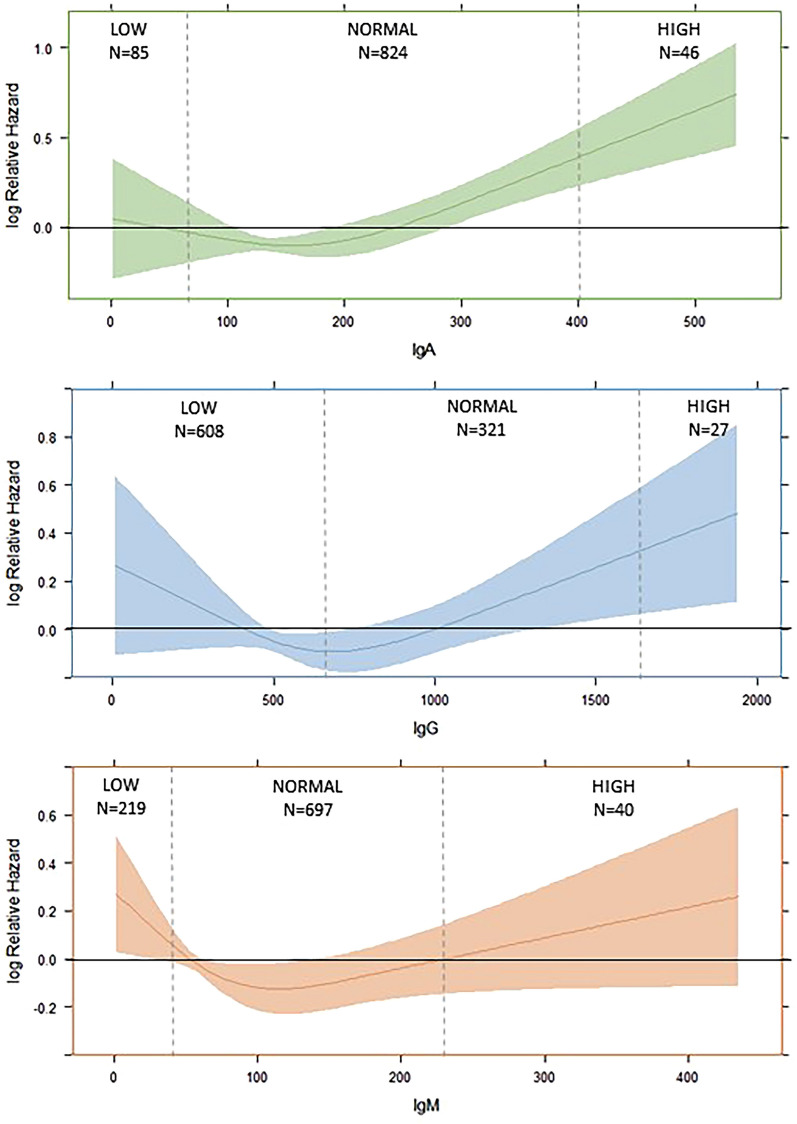
Splines for 90-day mortality, dotted line represents the inflection point

**Fig. 2:**
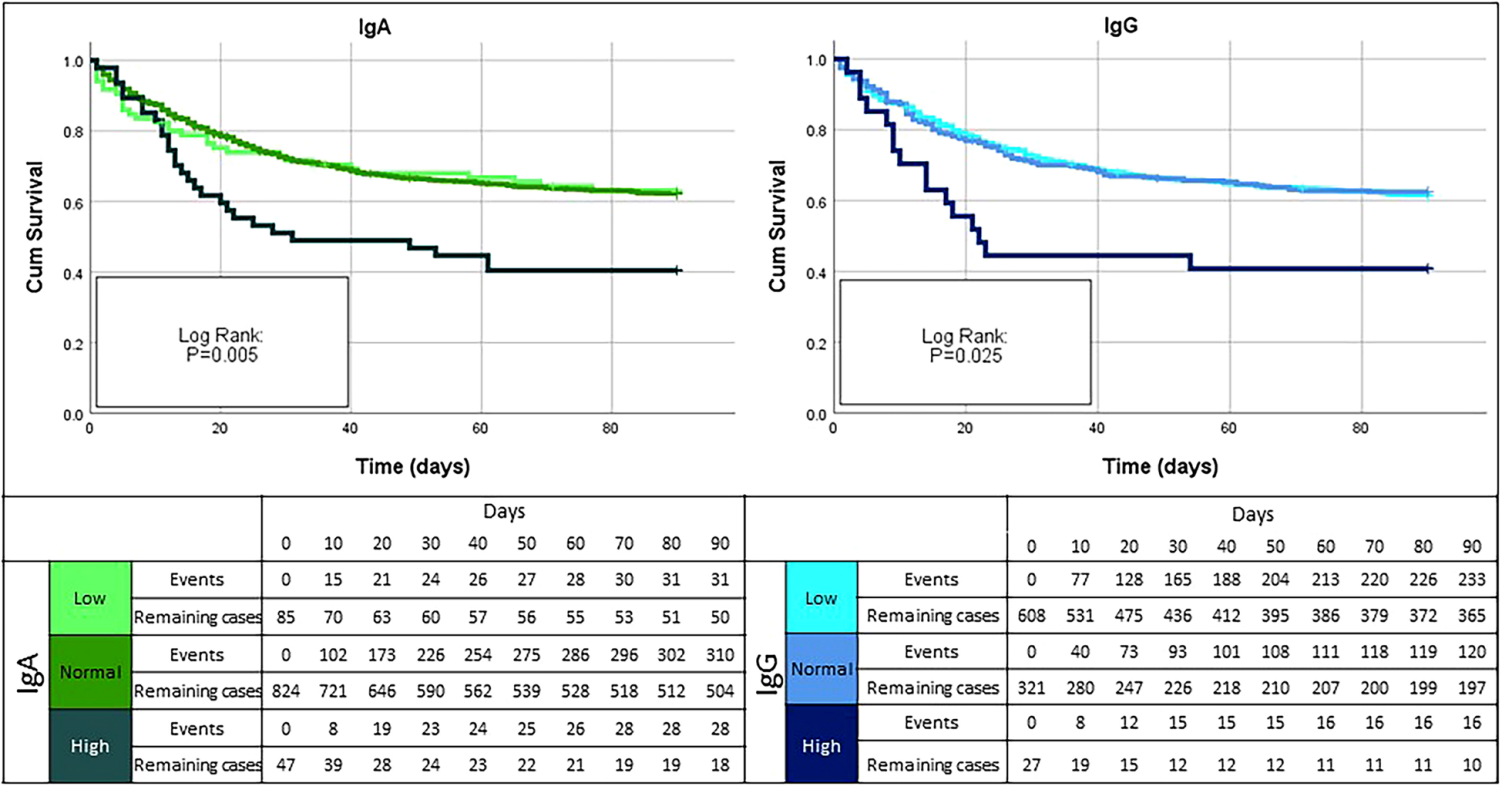
Kaplan–Meier analysis for IgA and IgG-groups and 90-day mortality

Patients with HIGH IgA had higher mortality (28-day mortality: 50.0%; 90-day mortality 60.9%) as compared to the other subgroups. Similar results were also observed for the HIGH IgG subgroup. In particular, for both IgA and IgG, 28-day mortality in the HIGH group was twofold higher as compared to LOW and NORMAL ranges (Additional file 1: Tables S1 and S2). No association between IgM and mortality at any time was found. These findings were confirmed by the restricted cubic splines (Fig. 1) and Kaplan–Meier curves analyses (Fig. 2).

The restricted cubic splines showed an inflection point for mortality at 150 mg/dL for IgA and 750 mg/dL for IgG, below these values we did not see any significant effect. Therefore, Cox proportional hazard survival analyses for z-transformed concentrations of IgA and IgG were performed only for patients above this cutoff (Table 2). Additional file 1: Table S5 depicts the baseline characteristics for survivors and non-survivors separately. Based upon this table the selection of variables to be included in multivariable regression was made: age, shock, baseline SOFA-score, creatinine, platelets, cardiovascular comorbidities, renal disease and COPD were included. IgA on day 1 had a significant predictive value for both 28-day and 90-day mortality (28-day mortality, HR: 1.50 (95% CI 1.18–1.92), *p* = 0.001 and 90-day mortality HR: 1.54 (95% CI 1.25–1.91), *p* < 0.001). IgG concentrations on day 1 showed similar results for 28-day (HR: 1.83 (95% CI 1.33–2.51), *p* < 0.001) and 90-day mortality (HR: 1.66 (95% CI 1.23–2.25), *p* = 0.001).

**Table 2:** Cox regression analysis for 28- and 90-day survival

Ig	Outcome	Model	HR	95% CI	P
IgA	28-day mortality (> 150 mg/dL)	**Univariate**	**1.24**	**1.07–1.43**	**0.004**
		**Multivariate**	**1.50**	**1.18–1.92**	**0.001**
	90-day mortality (> 150 mg/dL)	**Univariate**	**1.24**	**1.09–1.41**	**0.001**
		**Multivariate**	**1.54**	**1.25–1.91**	**6.3 × 10**^**–5**^
IgG	28-day mortality (> 750 mg/dL)	**Univariate**	**1.21**	**1.02–1.44**	**0.033**
		**Multivariate**	**1.83**	**1.33–2.51**	**1.8 × 10**^**–4**^
	90-day mortality (> 750 mg/dL)	Univariate	1.16	0.99–1.37	0.070
		**Multivariate**	**1.66**	**1.23–2.25**	**0.001**

Based upon the restricted cubic spline for IgA > 150 was included (*N* = 124) and for IgG > 750 (*N* = 294)

All values were Z-standardized—HR for 1 SD

Both multivariate Cox proportional hazards regression analyses were adjusted for clinical characteristics which were associated with 28- and 90-day mortality with a *p* value of < 0.0001: age, shock, baseline SOFA score, creatinine, platelets, cardiovascular comorbidities, renal comorbidities and COPD, see Additional file 1: Table S5

### Immunoglobulins and secondary infections

Of the 956 patients included in this study, 246 experienced a secondary infection. Lower levels of IgG at day 1 were associated with higher risk of secondary infections (median 533 IQR 406–772 vs median 600 IQR 452–842, *p* = 0.007). This difference was not observed for IgA and IgM.

## Discussion

In the largest cohort study of patients with severe sepsis or septic shock, we found that high levels of IgA and IgG on the first day of diagnosis were associated with a decreased 90-day survival. No association was found between IgM levels and survival. As such, the assessment of endogenous immunoglobulins could be a useful tool to identify septic patients at high risk of mortality.

The three classes of immunoglobulins act synergically in the immune response. IgG mainly controls secondary antibody responses, opsonization and complement activation, IgA works for the mucosal immunity, while the key functions of IgM are complement activation and primary antibody responses [27]. The association of immunoglobulin levels with patients’ survival is controversial [28]. Even if the majority of previous studies included a small number of patients, most of them reported that hypogammaglobulinemia was associated with a worse outcome [6, 7, 27]. In particular, Bermejo–Martin et al. [27] showed in 172 adult patients with severe sepsis or septic shock that low plasma concentrations of IgA, IgG and IgM were associated with reduced survival, which was not the case in ALBIOS. Those studies showed a correlation between low levels of IgG and mortality [29], but also with other clinical outcomes, such as vasopressor-free days and acute lung injury [6]. Differently from these findings, a recent meta-analysis, showed that the prevalence of hypogammaglobulinemia on the day of sepsis diagnosis is as high as 70%, but no specific sub-group of patients with a higher risk of death was identified [13]. Indeed, only in patients with total SOFA score < 8, the simultaneous presence of low levels of IgA, IgG and IgM was associated with higher mortality. More recently, results from the SBITs (Score-based immunoglobulin G therapy of patients with sepsis) study demonstrated that baseline hypogammaglobulinemia does not correlate with the survival in 543 patients with severe sepsis and septic shock. Moreover, patients with the highest IgG levels (fourth quartile, IgG > 1200 mg/dl) had a higher mortality compared to the reference quartile [22, 30].

In the cohort of the ALBIOS study, higher levels of IgA and IgG were associated with a higher risk of mortality. It is known that immunoglobulins play a crucial role in host response to infection, given their ability to neutralize endotoxins [31], to limit cytokines production [32], to increase serum bactericidal activity [33] and to attenuate the hyper activation of the complement cascade [34]. However, it is important also to consider a paradoxical effect of immune system activation. Higher levels of immunoglobulins could be a consequence of a hyper activation of the inflammatory response that can contribute to the paralysis of immune system during this critical state [30]. In addition, the association between IgA/IgG and mortality is independent of antibiotic therapy. In other words, the association with increased mortality of HIGH levels of IgA and IgG, cannot be explained by a different rate of appropriate antibiotic therapy.

In our study, IgM levels did not correlate with in-hospital mortality, although low IgM levels were associated with the occurrence of septic shock. In a previous study, Giamarellos-Bourbolis et al. found a correlation between IgM levels and mortality, highlighting that the concentration of IgM over time was greater in survivors than in non-survivors from septic shock [35]. Data from animal and in vitro studies suggest that IgM are released by a particular class of B lymphocytes belonging to the innate defence system, containing IgM in their cytoplasmic stores. In experimental sepsis models, depletion of B-lymphocytes leads to early death [36]. Moreover, patients with sepsis produced much less IgM than healthy volunteers [35–37]. Therefore, one could argue that during sepsis B lymphocytes may produce less IgM, so that patients’ necessity of adaptive immunity are compensated by circulating IgM. Once septic shock develops, circulating IgM are rapidly depleted and deficient production by B-lymphocytes cannot compensate for loss.

Recently, the analysis of clinical data from more than 20,000 patients, classified according to Sepsis-3 definition [1], allowed to define four phenotypes with different clinical characteristics [38]. Consistent differences in biomarker patterns and mortality rate were observed in the different phenotypes, and simulations suggested that these phenotypes may help understanding heterogeneity in the response to treatments. Four different genotypes were also identified by genome-wide gene expression profiles of blood from sepsis patients on admission to Intensive Care Unit for sepsis [39]. In addition, in this case, the four genotypes were characterized by different mortality rate and biomarkers profile, providing a tool for personalized patient management and novel criteria for selection of patients for trials. Therefore, the discrepancy observed when investigating the role of immunoglobulins in patient stratification and severity of the disease could be likely due to the heterogeneity of the population analysed without further classification. None of the included inflammatory biomarkers was associated with immunoglobulin levels, with the only exception of a significant inverse correlation of PTX3 with IgG and IgM levels and an association of PCSK9 to IgG. The specific inverse correlation of PTX3 with all 3 Igs, in the absence of any correlation of other inflammatory markers suggests an involvement of innate immunity, to which PTX3 pertains. The further classification of sepsis patients according to the genotypes defined by genome-wide gene expression profiles could possibly help to better understand the role of inflammatory molecules.

Several limitations are worth mentioning. First, we did not assay specific subtypes of immunoglobulins, which may have provided useful clues to the interpretation of the data. Second, we did not assess the time course of Ig’s in the whole cohort of patients, due to limitations in resources. Third, it was not possible to investigate the relation between the etiological agent of sepsis, where present, and the immunoglobulin time course.

In our study low baseline levels of IgG correlate with the risk of secondary infections after the first sepsis episodes. To our knowledge, this is a novel finding not previously reported. It is conceivable that during the acute phase of sepsis a “paralysis” of the immune system often occurs, in addition to the consumption and loss of immunoglobulins. A poor immunoglobulin reserve can result in a lack of immune control during the late or recovery sepsis phase, allowing some other infectious episodes.

## Conclusion

In this study, higher levels of IgG and IgA were associated with 90-day mortality in patients with sepsis or septic shock. Our data also suggest that the knowledge on pathophysiological mechanisms underlying the interaction of immunoglobulins during sepsis and its association with clinical outcomes are incomplete.

## Supplementary Information

**Additional file 1: Table S1. **Patient characteristics by IgA groups. **Table S2. **Patient characteristics by IgG groups.**Table S3. **Patient characteristics by IgM groups.**Table S4. **Plasma concentrations of IgA, IgG & IgM on day 1, 2 & 7 by day 1 levels.**Table S5. **Patient characteristics by 90-day survival.

## Data Availability

Data supporting this study and the ALBIOS study are not publicly available.

## Abbreviations

AIFA: Italian medicine agency
ALBIOS: Albumin Italian Outcome Septic Shock
BMI: Body Mass Index
CI 95%: 95% Confidence interval
EDTA: Ethylenediaminetetraacetic acid
HR: Hazard ratio
ICU: Intensive Care Unit
IgA: Immunoglobulin A
IgG: Immunoglobulin G
IgM: Immunoglobulin M
IQR: Interquartile range
IVIG: Intravenous immunoglobulins
LOQ: Limit of Quantitation
RCT: Randomized controlled trial
SBIT: Score-based immunoglobulin G therapy of patients with sepsis
SOFA: Sequential Organ Failure Assessment
