# Supplementary material for: Higher levels of IgA and IgG at sepsis onset are associated with higher mortality: results from the Albumin Italian Outcome Sepsis (ALBIOS) trial
Source: Ann Intensive Care. 2021 Nov 26;11:161. doi: 10.1186/s13613-021-00952-z (PMC8626546; doi:10.1186/s13613-021-00952-z)
Supplement: Supplementary file 1 — Additional file 1: Table S1. Patient characteristics by IgA groups. Table S2. Patient characteristics by IgG groups.Table S3. Patient characteristics by IgM groups.Table S4. Plasma concentrations of IgA, IgG & IgM on day 1, 2 & 7 by day 1 levels.Table S5. Patient characteristics by 90-day survival. [file 13613_2021_952_MOESM1_ESM.docx]

# **SUPPLEMENTAL DATA**

# **Higher levels of IgA and IgG at sepsis onset are associated with higher mortality: results from the Albumin Italian Outcome Sepsis (ALBIOS) trial.**

Laura Alagna^1*^, MD; Jennifer M.T.A. Meessen^2*^, PhD; Giacomo Bellani^3 4^, MD, PhD; Daniela Albiero^3^, MD; Pietro Caironi^5,6^, MD; Irene Principale^5^, MD; Luigi Vivona^7^, MD, Giacomo Grasselli^7,8^, MD, PhD; Francesca Motta^2^, BSc; Nicolò M. Agnelli^2^, BSc; Vieri Parrini^9^, MD; Stefano Romagnoli^10,11^, MD; Roberto Keim^12^, MD; Francesca Di Marzo Capozzi^13^, MSc; Fabio S. Taccone^14^, MD, PhD; Walter Taccone^13^, MD; Alessandra Bandera^1,7^, MD; Andrea Cortegiani^15,16^, MD; Roberto Latini^2^, MD.

^*shared first authorship^

**Supplementary Table 1** – Patient characteristics by IgA groups.

**Supplementary Table 2** – Patient characteristics by IgG groups.

**Supplementary Table 3** – Patient characteristics by IgM groups.

**Supplementary Table 4 -** Plasma concentrations of IgA, IgG & IgM on day 1, 2 & 7 by day 1 levels.

**Supplementary Table 5.** Patient characteristics by 90-day survival.

**Supplementary Table 1.** Patient characteristics by IgA groups.

|  | | | |  | **IgA** | | | P |
| --- | --- | --- | --- | --- | --- | --- | --- | --- |
|  |  |  |  | Total  N=956* | Low range  1-70  N=85 | Normal range  70-400  N=824 | High range  400-767  N=47 |  |
| IgA mg/dL | | Day 1 (N=955) | | 161.0 [110.0-227.0] | 49.0 [34.0-59.0] | 168.5 [120.3-221.0] | 469.0 [443.0-526.5] | - |
|  |  | Day 2 (N=150) | | 185.6 [133.0-262.8] | 63.5 [59.3-80.8] | 188.0 [142.0-248.5] | 458.0 [394.5-515.8] | **1.3x10^-11^** |
|  |  | Day 7 (N=150) | | 240.5 [167.5-324.5] | 110.5 [88.5-154.3] | 244.0 [174.0-315.8] | 444.0 [418.5-582.5] | **2.2x10^-9^** |
| IgG mg/dL | | Day 1 (N=956) | | 588 [438-816] | 341 [201-473] | 592 [455-797] | 1166 [923-1424] | **1.5x10^-30^** |
|  |  | Day 2 (N=150) | | 591 [460-787] | 405 [248-558] | 588 [475-785] | 1056 [744-1329] | **9.0x10^-6^** |
|  |  | Day 7 (N=150) | | 739 [555-980] | 567 [406-717] | 747 [558-980] | 1060 [686-1382] | **0.003** |
| IgM mg/dL | | Day 1 (N=956) | | 66.0 [42.0-109.0] | 34.0 [1.0-76.3] | 66.0 [44.0-106.8] | 118.0 [74.0-170.0] | **2.2x10^-13^** |
|  |  | Day 2 (N=150) | | 57.5 [38.0-87.3] | 34.0 [10.0-44.8] | 57.5 [40.0-84.0] | 134.5[114.5-209.5] | **2.0x10^-5^** |
|  |  | Day 7 (N=150) | | 85.0 [56.8-132.0] | 79.5 [63.3-155.8] | 83.0 [54.0-131.3] | 133.0 [114.5-214.5] | **0.043** |
| Randomized to albumin | | | | 473 (49.5%) | 34 (40.0%) | 420 (51.0%) | 29 (61.7%) | **0.045** |
| Age | | | years | 66.6 ± 14.7 | 64.6 ± 15.1 | 66.8 ± 14.8 | 66.6 ± 13.0 | 0.345 |
| Sex | | | female | 395 (41.3%) | 48 (56.5%) | 334 (40.5%) | 13 (27.7%) | **0.003** |
| BMI | | | kg/m^2^ | 26.4 ± 5.6 | 25.2 ± 4.8 | 26.4 ± 5.5 | 28.3 ± 7.9 | **0.019** |
| Shock | | | | 538 (56.3%) | 49 (57.6%) | 469 (56.9%) | 20 (42.6%) | 0.150 |
| Baseline SOFA score | | | | 8.9 ± 2.4 | 8.4 ± 2.5 | 8.9 ± 2.4 | 8.6 ± 2.6 | 0.491 |
| Mean arterial pressure | | | mmHg | 74.6 ± 14.9 | 73.3 ± 167 | 74.8 ± 14.9 | 74.0 ± 12.4 | 0.586 |
| Heart Rate | | | bpm | 104 ± 21 | 107 ± 21 | 104 ± 21 | 97 ± 20 | 0.073 |
| Lactate | | | mmol/L | 2.72 [1.70-4.69] | 2.79 [1.50-4.65] | 2.80 [1.80-4.75] | 2.00 [1.25-3.65] | 0.218 |
| Creatinine | | | mg/dL | 1.50 [0.90-2.50] | 1.30 [0.90-2.10] | 1.50 [0.90-2.60] | 1.10 [0.80-1.90] | **0.008** |
| Platelet counts | | | x10^3^/mm^3^ | 166 [102-237] | 139 [87-231] | 167 [105-237] | 162 [89-251] | 0.445 |
| White blood cells | | | x10^3^/mm^3^ | 12.0 [5.7-18.6] | 8.7 [3.4-17.5] | 12.1 [5.9-18.6] | 13.2 [8.9-19.4] | **0.006** |
| Hemoglobin | | | g/dL | 10.9 [9.9-12.2] | 10.8 [9.8-12.7] | 11.0 [9.9-12.3] | 10.7 [9.5-11.8] | 0.601 |
| Central venous O_2_ saturation | | | % | 75 [68-80] | 76 [70-80] | 75 [68-80] | 76 [71-82] | 0.636 |
| Comorbidities | Immunocompromised | | | 124 (13.0%) | 24 (28.2%) | 94 (11.4%) | 6 (12.8%) | **6.3x10^-5^** |
|  | AIDS | | | 11 (1.2%) | 0 | 10 (1.2%) | 1 (2.1%) | 0.493 |
|  | Onco-haematologic | | | 35 (3.7%) | 12 (14.1%) | 21 (2.5%) | 2 (4.3%) | **4.4x10^-7^** |
|  | Metastatic tumors | | | 35 (3.7%) | 2 (2.4%) | 33 (4.0%) | 0 | 0.290 |
|  | Cardiovascular | | | 164 (17.2%) | 6 (7.1%) | 147 (17.8%) | 11 (23.4%) | **0.022** |
|  | Liver disease | | | 14 (1.5%) | 1 (1.2%) | 12 (1.5%) | 1 (2.1%) | 0.908 |
|  | Renal disease | | | 42 (4.4%) | 2 (2.4%) | 39 (4.7%) | 1 (2.1%) | 0.440 |
|  | COPD | | | 120 (12.6%) | 5 (5.9%) | 106 (12.9%) | 9 (19.1%) | 0.068 |
| Location of infection | Lung | | | 326 (34.1%) | 25 (29.4%) | 281 (34.1%) | 20 (42.6%) | 0.290 |
|  | Abdomen | | | 329 (34.4%) | 33 (38.8%) | 287 (34.8%) | 9 (19.1%) |  |
|  | Genitourinay Tract | | | 89 (9.3%) | 6 (7.1%) | 80 (9.7%) | 3 (6.4%) |  |
|  | Other | | | 107 (11.2%) | 10 (11.8%) | 88 (10.7%) | 9 (19.1%) |  |
|  | Multiple | | | 105 (11.0%) | 11 (12.9%) | 88 (10.7%) | 6 (12.8%) |  |
| Biomarkers  at day 1 | Presepsin | | | 949 [494-1898] | 1018 [542-2082] | 939 [491-1884] | 862 [418-1930] | 0.706 |
|  | Pentraxin-3 | | | 71.9 [32.9-186.3] | 97.9 [34.9-224.6] | 71.9 [33.0-187.8] | 49.3 [27.1-103.3] | 0.073 |
|  | PCSK9 (ng/mL) | | | 277.7 [182.2-452.1] | 289.3 [201.8-498.2] | 278.4 [182.8-453.1] | 234.3 [160.9-347.6] | 0.063 |
|  | Resistin (ng/mL) | | | 192.7 [90.3-452.3] | 220.3 [84.8-427.3] | 189.2 [90.1-461.7] | 236.6 [97.7-422.7] | 0.903 |
|  | MPO (ng/mL) | | | 128.1 [69.8-247.2] | 119.0 [66.6-242.1] | 128.6 [70.0-258.3] | 135.1 [81.4-321.9] | 0.778 |
|  | OPN (ng/mL) | | | 563 [339-929] | 564 [357-922] | 565 [341-938] | 420 [264-856] | 0.584 |
| Appropriateness of antibiotic therapy according to site culture (%) | | | | 421/557 (75.6%) | 42/52 (80.8%) | 355/476 (74.6%) | 24/29 (82.8%) | 0.401 |
| 28 day mortality | | | | 263 (27.5%) | 22 (25.9%) | 218 (26.5%) | 23 (48.9%) | **0.003** |
| 90 day mortality | | | | 369 (38.6%) | 31 (36.5%) | 310 (37.6%) | 28 (59.6%) | **0.010** |
| P-value for Chi-square or Kruskal-Wallis test for differences between groups of IgA value. * for one patient the IgA level at baseline is missing. | | | | | | | | |

**Supplementary Table 2.** Patient characteristics by IgG groups.

|  | | | | Total  N=956 | **IgG** | | | P |
| --- | --- | --- | --- | --- | --- | --- | --- | --- |
|  |  |  |  |  | Low range  10-700  N=608 | Normal range  700-1600  N=321 | High range  1600-3170  N=27 |  |
| IgA mg/dL | | Day 1 (N=955) | | 161.0 [110.0-227.0] | 133.0 [93.0-185.0] | 22.0 [164.0-295.5] | 315.0 [207.0-443.0] | **2.3x10^-46^** |
|  |  | Day 2 (N=150) | | 185.6 [133.0-262.8] | 165.0 [119.8-214.8] | 257.5 [172.5-364.0] | 286.0 [170.3-465.5] | **8.2x10^-7^** |
|  |  | Day 7 (N=150) | | 240.5 [167.5-324.5] | 224.0 [160.8-298.3] | 263.0 [192.3-385.3] | 206.0 [166.0-525.8] | **0.034** |
| IgG mg/dL | | Day 1 (N=956) | | 588 [438-816] | 477 [372-572] | 900 [776-1076] | 1769 [1661-2003] | **-** |
|  |  | Day 2 (N=150) | | 591 [460-787] | 504 [409-594] | 842 [748-1045] | 1609 [1399-1905] | **2.1x10^-20^** |
|  |  | Day 7 (N=150) | | 739 [555-980] | 622 [509-812] | 921 [782-1144] | 1177 [1047-1835] | **3.9x10^-9^** |
| IgM mg/dL | | Day 1 (N=956) | | 66.0 [42.0-109.0] | 53.0 [34.0-81.0] | 92.0 [62.0-139.0] | 130.0 [73.0-170.0] | **5.1x10^-32^** |
|  |  | Day 2 (N=150) | | 57.5 [38.0-87.3] | 50.0 [33.0-74.3] | 77.5 [56.0-115.0] | 132.5 [118.8-197.3] | **2.8x10^-5^** |
|  |  | Day 7 (N=150) | | 85.0 [56.8-132.0] | 79.5 [52.8-127.3] | 90.5 [61.0-132.0] | 133.5 [96.0-210.0] | 0.204 |
| Randomized to albumin | | | | 473 (49.5%) | 319 (52.5%) | 138 (43.0%) | 16 (59.3%) | **0.013** |
| Age | | | years | 66.6 ± 14.7 | 67.5 ±14.3 | 65.0 ± 15.2 | 66.0 ± 16.8 | 0.054 |
| Sex | | | female | 395 (41.3%) | 274 (45.1%) | 115 (35.8%) | 6 (22.2%) | **0.003** |
| BMI | | | kg/m2 | 26.4 ± 5.6 | 26.0 ± 5.3 | 27.2 ± 6.1 | 27.0 ± 5.0 | **0.015** |
| Shock | | | | 538 (56.3%) | 361 (59.4%) | 163 (50.8%) | 14 (51.9%) | **0.038** |
| Baseline SOFA score | | | | 8.9 ± 2.4 | 8.8 ± 2.4 | 9.0 ± 2.5 | 9.5 ± 2.7 | 0.609 |
| Mean arterial pressure | | | mmHg | 74.6 ± 14.9 | 73.7 ± 15.0 | 76.0 ± 14.8 | 77.7 ± 15.7 | 0.059 |
| Heart Rate | | | bpm | 104 ± 21 | 105 ± 20 | 102 ± 22 | 97 ± 16 | **0.040** |
| Lactate | | | mmol/L | 2.72 [1.70-4.69] | 2.89 [1.72-4.65] | 2.55 [1.60-4.64] | 2.80 [2.05-4.85] | 0.794 |
| Creatinine | | | mg/dL | 1.50 [0.90-2.50] | 1.40 [0.90-2.50] | 1.65 [0.90-2.70] | 1.35 [0.90-2.73] | 0.435 |
| Platelets | | | x10^3^/mm^3^ | 166 [102-237] | 166 [106-230] | 167 [97-248] | 142 [80-233] | 0.838 |
| White blood cells | | | x10^3^/mm^3^ | 12.0 [5.7-18.6] | 11.1 [4.7-17.7] | 13.4 [8.1-19.5] | 12.4 [7.4-19.1] | **3.5x10^-4^** |
| Hemoglobin | | | g/dL | 10.9 [9.9-12.2] | 11.0 [9.9-12.3] | 10.9 [10.0-12.0] | 10.3 [9.5-12.2] | 0.819 |
| Central O_2_ saturation | | | % | 75 [68-80] | 74 [68-80] | 77 [70-81] | 80 [67-81] | 0.090 |
| Comorbidities | Immunocompromized | | | 124 (13.0%) | 85 (14.0%) | 31 (9.7%) | 8 (29.6%) | **0.006** |
|  | AIDS | | | 11 (1.2%) | 6 (1.0%) | 2 (0.6%) | 3 (11.1%) | **5.0x10^-6^** |
|  | Onco-haematologic | | | 35 (3.7%) | 24 (3.9%) | 8 (2.5%) | 3 (11.1%) | 0.060 |
|  | Metastatic tumors | | | 35 (3.7%) | 26 (4.3%) | 8 (2.5%) | 1 (3.7%) | 0.388 |
|  | Cardiovascular | | | 164 (17.2%) | 95 (15.6%) | 64 (19.9%) | 5 (18.5%) | 0.248 |
|  | Liver disease | | | 14 (1.5%) | 8 (1.3%) | 4 (1.2%) | 2 (7.4%) | **0.033** |
|  | Renal disease | | | 42 (4.4%) | 22 (3.6%) | 20 (6.2%) | 0 | 0.096 |
|  | COPD | | | 120 (12.6%) | 68 (11.2%) | 48 (15.0%) | 4 (14.8%) | 0.241 |
| Location of infection | Lung | | | 326 (34.1%) | 193 (31.7%) | 118 (36.8%) | 15 (55.6%) | **1.0x10^-6^** |
|  | Abdomen | | | 329 (34.4%) | 251 (41.3%) | 73 (22.7%) | 5 (18.5%) |  |
|  | Genitourinay Tract | | | 89 (9.3%) | 49 (8.1%) | 39 (12.1%) | 1 (3.7%) |  |
|  | Other | | | 107 (11.2%) | 61 (10.0%) | 44 (13.7%) | 2 (7.4%) |  |
|  | Multiple | | | 105 (11.0%) | 54 (8.9%) | 47 (14.6%9 | 4 (14.8%) |  |
| Biomarkers  at day 1 | Presepsin | | | 949 [494-1898] | 952 [511-1780] | 934 [426-2078] | 923 [538-2109] | 0.913 |
|  | Pentraxin-3 | | | 71.9 [32.9-186.3] | 89.0 [35.8-213.3] | 51.1 [27.9-144.8] | 59.8 [25.8-129.9] | **3.2x10^-5^** |
|  | PCSK9 (ng/mL) | | | 277.7 [182.2-452.1] | 299.2 [197.2-492.3] | 253.4 [169.8-413.4] | 265.6 [179.3-417.7] | **0.001** |
|  | Resistin (ng/mL) | | | 192.7 [90.3-452.3] | 206.3 [93.5-459.6] | 174.1 [88.2-436.7] | 138.1 [62.5-474.3] | 0.300 |
|  | MPO (ng/mL) | | | 128.1 [69.8-247.2] | 124.3 [68.1-355.4] | 131.7 [70.1-271.8] | 138.9 [81.1-263.1] | 0.584 |
|  | OPN (ng/mL) | | | 563 [339-929] | 577 [351-942] | 518 [317-882] | 466 [254-994] | 0.266 |
| Appropriateness of antibiotic therapy according to site culture(%) | | | | 421/557 (75.6%) | 273/357 (76.5%) | 137/186 (73.7%) | 11/14 (78.6%) | 0.743 |
| 28 day mortality | | | | 263 (27.5%) | 157 (25.8%) | 91 (28.3%) | 15 (55.6%) | **0.003** |
| 90 day mortality | | | | 369 (38.6%) | 233 (38.3%) | 120 (37.4%) | 16 (59.3%) | 0.079 |
| P-value for Chi2 or Kruskal-Wallis test for differences between groups of IgG value. | | | | | | | | |

**Supplementary Table 3.** Patient characteristics by IgM groups.

|  | | | | Total  N=956 | **IgM** | | | P |
| --- | --- | --- | --- | --- | --- | --- | --- | --- |
|  |  |  |  |  | Low range  1-40  N=219 | Normal range  40-230  N=697 | High range  230-864  N=40 |  |
| IgA mg/dL | | Day 1 (N=955) | | 161.0 [110.0-227.0] | 120.0 [75.0-186.0] | 174.0 [122.0-238.0] | 168.0 [119.0-282.0] | **7.1x10^-13^** |
|  |  | Day 2 (N=150) | | 185.6 [133.0-262.8] | 146.0 [84.0-203.0] | 197.0 [145.3-283.8] | 235.0 * | **2.0x10^-4^** |
|  |  | Day 7 (N=150) | | 240.5 [167.5-324.5] | 219.0 [137.0-277.0] | 245.5 [174.0-332.8] | 270.0 * | **0.025** |
| IgG mg/dL | | Day 1 (N=956) | | 588 [438-816] | 435 [308-559] | 653 [480-875] | 703 [542-1024] | **2.7x10^-29^** |
|  |  | Day 2 (N=150) | | 591 [460-787] | 448 [308-521] | 675 [513-845] | 600 * | **1.2x10^-8^** |
|  |  | Day 7 (N=150) | | 739 [555-980] | 526 [413-744] | 826 [620-1019] | 641 * | **6.3x10^-7^** |
| IgM mg/dL | | Day 1 (N=956) | | 66.0 [42.0-109.0] | 27.0 [1.0-34.0] | 78.0 [56.0-115.0] | 337.0 [268.0-434.0] | **-** |
|  |  | Day 2 (N=150) | | 57.5 [38.0-87.3] | 31.0 [23.0-38.0] | 73.5 [56.0-108.0] | 320.0 * | **2.7x10^-19^** |
|  |  | Day 7 (N=150) | | 85.0 [56.8-132.0] | 45.0 [31.0-67.0] | 104.5 [74.0-135.8] | 227.0 * | **5.3x10^-12^** |
| Randomized to albumin | | | | 473 (49.5%) | 122 (55.7%) | 334 (47.9%) | 17 (42.5%) | 0.088 |
| Age | | | years | 66.6 ± 14.7 | 70.7 ± 11.6 | 65.3 ± 15.2 | 67.6 ± 17.5 | **2.9x10^-5^** |
| Sex | | | female | 395 (41.3%) | 88 (40.2%) | 290 (41.6%) | 17 (42.5%) | 0.922 |
| BMI | | | kg/m^2^ | 26.4 ± 5.6 | 26.4 ± 5.1 | 26.4 ± 5.8 | 26.3 ± 4.0 | 0.672 |
| Shock | | | | 538 (56.3%) | 137 (62.6%) | 385 (55.2%) | 16 (40.0%) | **0.017** |
| Baseline SOFA score | | | | 8.9 ± 2.4 | 8.8 ± 2.4 | 8.9 ± 2.4 | 7.6 ± 1.6 | 0.279 |
| Mean arterial pressure | | | mmHg | 74.6 ± 14.9 | 72.1 ± 14.6 | 75.3 ± 15.1 | 76.4 ± 13.0 | **0.010** |
| Heart Rate | | | bpm | 104 ± 21 | 105 ± 21 | 104 ± 21 | 99 ± 20 | 0.210 |
| Lactate | | | mmol/L | 2.72 [1.70-4.69] | 2.90 [1.68-4.93] | 2.70 [1.70-4.60] | 1.80 [1.40-3.20] | 0.237 |
| Creatinine | | | mg/dL | 1.50 [0.90-2.50] | 1.50 [1.05-2.70] | 1.40 [0.90-2.50] | 1.50 [0.90-2.60] | 0.101 |
| Platelets | | | x10^3^/mm^3^ | 166 [102-237] | 164 [99-224] | 166 [104-242] | 171 [74-254] | 0.798 |
| White blood cells | | | x10^3^/mm^3^ | 12.0 [5.7-8.6] | 9.4 [4.3-18.1] | 12.3 [6.4-18.6] | 11.4 [6.7-18.8] | **0.009** |
| Hemoglobin | | | g/dL | 10.9 [9.9-12.2] | 11.0 [10.0-12.5] | 10.9 [9.9-12.1] | 10.1 [9.9-11.9] | 0.420 |
| Central O_2_ saturation | | | % | 75 [68-80] | 74 [67-81] | 76 [69-80] | 68 [51-78] | 0.161 |
| Comorbidities | Immunocompromized | | | 124 (13.0%) | 33 (15.1%) | 86 (12.3%) | 5 (12.5%) | 0.575 |
|  | AIDS | | | 11 (1.2%) | 3 (1.4%) | 7 (1.0%) | 1 (2.5%) | 0.649 |
|  | Onco-haematologic | | | 35 (3.7%) | 14 (6.4%) | 20 (2.9%) | 1 (2.5%) | **0.049** |
|  | Metastatic tumors | | | 35 (3.7%) | 5 (2.3%) | 30 (4.3%) | 0 | 0.172 |
|  | Cardiovascular | | | 164 (17.2%) | 33 (15.159 | 122 (17.5%) | 9 (22.5%) | 0.464 |
|  | Liver disease | | | 14 (1.5%) | 3 (1.4%) | 10 (1.4%) | 1 (2.5%) | 0.854 |
|  | Renal disease | | | 42 (4.4%) | 12 (5.5%) | 28 (4.0%) | 2 (5.0%) | 0.642 |
|  | COPD | | | 120 (12.6%) | 31 (14.2%) | 84 (12.1%) | 5 (12.5%) | 0.715 |
| Location of infection | Lung | | | 326 (34.1%) | 66 (30.1%) | 235 (33.7%) | 25 (62.5%) | **0.001** |
|  | Abdomen | | | 329 (34.4%) | 94 (42.9%) | 229 (32.9%) | 6 (15.0%) |  |
|  | Genitourinay Tract | | | 89 (9.3%) | 16 (7.3%) | 73 (10.5%) | 0 |  |
|  | Other | | | 107 (11.2%) | 20 (9.1%) | 83 (11.9%) | 4 (10.0%) |  |
|  | Multiple | | | 105 (11.0%) | 23 (10.5%) | 77 (11.0%) | 5 (12.5%) |  |
| Biomarkers  at day 1 | Presepsin | | | 949 [494-1898] | 980 [541-2153] | 923 [484-1870] | 833 [452-2099] | 0.376 |
|  | Pentraxin-3 | | | 71.9 [32.9-186.3] | 99.4 [34.7-227.5] | 64.3 [32.0-176.7] | 49.4 [26.1-130.0] | **0.017** |
|  | PCSK9 (ng/mL) | | | 277.7 [182.2-452.1] | 298.6 [197.5-495.3] | 272.3 [178.0-436.1] | 285.9 [181.1-426.3] | 0.076 |
|  | Resistin (ng/mL) | | | 192.7 [90.3-452.3] | 255.1 [90.1-533.6] | 184.0 [89.7-434.4] | 154.2 [99.2-398.9] | 0.297 |
|  | MPO (ng/mL) | | | 128.1 [69.8-247.2] | 117.8 [65.7-255.4] | 128.5 [70.4-263.0] | 134.9 [83.8-255.5] | 0.512 |
|  | OPN (ng/mL) | | | 563 [339-929] | 600 [349-1004] | 552 [331-936] | 466 [320-661] | 0.152 |
| Appropriateness of antibiotic therapy according to site culture(%) | | | | 421/557 (75.6%) | 96/130 (73.8%) | 308/406 (75.9%) | 17/21 (81.0%) | 0.757 |
| 28 day mortality | | | | 263 (27.5%) | 69 (31.5%) | 181 (26.0%) | 13 (32.5%) | 0.214 |
| 90 day mortality | | | | 369 (38.6%) | 97 (44.3%) | 256 (36.7%) | 16 (40.0%) | 0.132 |
| P-value for Chi2 or Kruskal-Wallis test for differences between groups of IgM value.  * Value available only for 2 patients | | | | | | | | |

**Supplemental Table 4.** Plasma concentrations of IgA, IgG and IgM on day 1, 2 and 7 by study treatment.

|  | Time | Total concentration  N=150 | Albumin treatment  n=80 | Crystalloids  n=70 | P |
| --- | --- | --- | --- | --- | --- |
| IgA mg/dL | Day 1 | 183.0 [125.0-247.0] | 181.5 [122.5-233.3] | 186.0 [137.0-287.0] | 0.184 |
|  | Day 2 | 185.6 [133.0-262.8] | 181.5 [132.3-241.0] | 186.5 [136.0-287.3] | 0.229 |
|  | Day 7 | 240.5 [167.5-324.5] | 232.5 [158.5-311.0] | 274.5 [180.5-337.5] | 0.130 |
| IgG mg/dL | Day 1 | 610.5 [446.0-776.8] | 566.0 [421.5-768.8] | 635.5 [451.0-797.8] | 0.284 |
|  | Day 2 | 591 [460-787] | 574.5 [433.5-783.5] | 611.5 [477.5-809.5] | 0.224 |
|  | Day 7 | 739 [555-980] | 736.5 [526.0-981.3] | 744.0 [593.5-957.8] | 0.282 |
| IgM mg/dL | Day 1 | 55.0 [37.0-92.0] | 55.0 [36.3-92.8] | 55.0 [37.8-82.3] | 0.953 |
|  | Day 2 | 57.5 [38.0-87.3] | 56.0 [38.5-102.5] | 63.5 [37.3-86.3] | 0.888 |
|  | Day 7 | 85.0 [56.8-132.0] | 86.5 [58.8-132.8] | 83.0 [55.8-131.3] | 0.746 |
| *P value for Mann-Whitney test to assess differences between treatment groups. | | | | | |

**Supplementary Table 5.** Patient characteristics by 90-day survival.

|  | | | | Total  N=956 | **Survival** | | P |
| --- | --- | --- | --- | --- | --- | --- | --- |
|  |  |  |  |  | Surivor  N=587 | Dead within 90 days  N=369 |  |
| IgA mg/dL | | Day 1 (N=955) | | 161.0 [110.0-227.0] | 159.0 [110.0-217.0] | 164.0 [111.3-254.8] | **0.028** |
|  |  | Day 2 (N=150) | | 185.6 [133.0-262.8] | 174.0 [128.5-207] | 203.0 [138.5-285.5] | **0.032** |
|  |  | Day 7 (N=150) | | 240.5 [167.5-324.5] | 221.0 [158.0-287.5] | 46.0 [176.0-334.5] | 0.079 |
| IgG mg/dL | | Day 1 (N=956) | | 588 [438-816] | 586.0 [439.0-792.0] | 592.0 [436.0-848.0] | 0.721 |
|  |  | Day 2 (N=150) | | 591 [460-787] | 545.0 [419.5-703.0] | 614.0 [490.0-869.5] | **0.012** |
|  |  | Day 7 (N=150) | | 739 [555-980] | 717.0 [527.0-889.5] | 778.0 [587.5-1008.5] | 0.072 |
| IgM mg/dL | | Day 1 (N=956) | | 66.0 [42.0-109.0] | 66.0 [43.0-109.0] | 64.0 [37.0-105.3] | 0.155 |
|  |  | Day 2 (N=150) | | 57.5 [38.0-87.3] | 52.0 [38.0-74.5] | 66.0 [39.0-102.0] | 0.092 |
|  |  | Day 7 (N=150) | | 85.0 [56.8-132.0] | 79.0 [56.0-120.5] | 90.0 [56.5-135.0] | 0.321 |
| Randomized to albumin | | | | 473 (49.5%) | 288 (49.1%) | 185 (50.1%) | 0.747 |
| **Age** | | | years | 66.6 ± 14.7 | 63.3 ± 15.2 | 71.8 ± 12.3 | **1.1x10^-19^** |
| **Sex** | | | female | 395 (41.3%) | 258 (44.0%) | 137 (37.1%) | **0.037** |
| **BMI** | | | kg/m^2^ | 26.4 ± 5.6 | 26.7 ± 5.6 | 26.0 ± 5.6 | **0.025** |
| **Shock** | | | | 538 (56.3%) | 304 (51.8%) | 234 (63.4%) | **4.2x10^-4^** |
| **Baseline SOFA score** | | | | 8.9 ± 2.4 | 8.5 ± 2.5 | 9.5 ± 2.2 | **1.5x10^-4^** |
| **Mean arterial pressure** | | | mmHg | 74.6 ± 14.9 | 75.6 ± 14.8 | 73.1 ± 15.1 | **0.017** |
| Heart Rate | | | bpm | 104 ± 21 | 103 ± 20 | 105 ± 21 | 0.321 |
| Lactate | | | mmol/L | 2.72 [1.70-4.69] | 2.70 [1.60-4.50] | 2.80 [1.90-4.82] | 0.255 |
| **Creatinine** | | | mg/dL | 1.50 [0.90-2.50] | 1.30 [0.80-2.20] | 1.90 [1.10-2.93] | **2.2x10^-9^** |
| **Platelets** | | | x10^3^/mm^3^ | 166 [102-237] | 177 [114-248] | 144 [83-212] | **6.0x10^-6^** |
| White blood cells | | | x10^3^/mm^3^ | 12.0 [5.7-8.6] | 12.0 [5.9-19.1] | 11.7 [5.3-17.6] | 0.262 |
| Hemoglobin | | | g/dL | 10.9 [9.9-12.2] | 10.9 [9.9-12.1] | 11.0 [9.7-12.3] | 0.700 |
| Central O_2_ saturation | | | % | 75 [68-80] | 75 [68-80] | 76 [68-81] | 0.641 |
| Comorbidities | **Immunocompromized** | | | 124 (13.0%) | 62 (10.6%) | 62 (16.8%) | **0.005** |
|  | AIDS | | | 11 (1.2%) | 6 (1.0%) | 5 (1.4%) | 0.638 |
|  | **Onco-haematologic** | | | 35 (3.7%) | 12 (2.0%) | 23 (6.2%) | **0.001** |
|  | Metastatic tumors | | | 35 (3.7%) | 19 (3.2%) | 16 (4.3%) | 0.378 |
|  | **Cardiovascular** | | | 164 (17.2%) | 70 (11.9%) | 94 (25.5%) | **6.3x10^-8^** |
|  | **Liver disease** | | | 14 (1.5%) | 5 (0.9%) | 9 (2.4%) | **0.047** |
|  | **Renal disease** | | | 42 (4.4%) | 15 (2.6%) | 27 (7.3%) | **4.7x10^-4^** |
|  | **COPD** | | | 120 (12.6%) | 53 (9.0%) | 67 (18.2%) | **3.4x10^-5^** |
| Location of infection | **Lung** | | | 326 (34.1%) | 188 (32.0%) | 138 (37.4%) | **0.005** |
|  | **Abdomen** | | | 329 (34.4%) | 210 (35.8%) | 119 (32.2%) |  |
|  | **Genito-urinary Tract** | | | 89 (9.3%) | 69 (11.8%) | 20 (5.4%) |  |
|  | **Other** | | | 107 (11.2%) | 62 (10.6%) | 45 (12.2%) |  |
|  | **Multiple** | | | 105 (11.0%) | 58 (9.9%) | 47 (12.7%) |  |
| Biomarkers  at day 1 | **Presepsin** | | | 949 [494-1898] | 732 [425-1272] | 1397 [699-2817] | **8.6x10^-21^** |
|  | **Pentraxin-3** | | | 71.9 [32.9-186.3] | 55.9 [29.1-161.6] | 101.7 [39.2-229.6] | **4.0x10^-6^** |
|  | **PCSK9 (ng/mL)** | | | 277.7 [182.2-452.1] | 294.6 [200.8-453.4] | 254.6 [169.2-433.9] | **0.004** |
|  | **Resistin (ng/mL)** | | | 192.7 [90.3-452.3] | 171.0 [87.5-400.2] | 240.2 [97.2-543.2] | **2.7x10^-4^** |
|  | **MPO (ng/mL)** | | | 128.1 [69.8-247.2] | 118.7 [68.6-227.8] | 143.5 [72.6-350.5] | **0.002** |
|  | **OPN (ng/mL)** | | | 563 [339-929] | 507.8 [314.0-818.0] | 642 [382-1129] | **1.1x10^-5^** |
| Appropriateness of antibiotic therapy according to site culture(%) | | | | 421/557 (75.6%) | 269 (80.3%) | 152 (68.5%) | **0.001** |
| P-value for Chi2 or Kruskal-Wallis test for differences between survivor and those deceased within 90 days. | | | | | | | |
